# Addiction to work and factors relating to this: a cross-sectional study on doctors in the state of Paraíba

**DOI:** 10.1590/1516-3180.2016.0312250417

**Published:** 2017-09-28

**Authors:** Walter Fernandes Azevedo, Lígia Andrade da Silva Telles Mathias

**Affiliations:** I MD, MSc. Doctoral Student, Faculdade de Ciências Médicas da Santa Casa de São Paulo (FCMSCSP), São Paulo; Anesthesiologist, Hospital Universitário Lauro Wanderley (HULW); and Adjunct Professor, Medical School, Universidade Federal da Paraíba (UFPB), João Pessoa (PB), Brazil.; II MD, MSc, PhD. Adjunct Professor, Medical School, Faculdade de Ciências Médicas da Santa Casa de São Paulo (FCMSCSP), São Paulo (SP), Brazil.

**Keywords:** Work, Behavior, addictive, Risk factors, Medicine, Health

## Abstract

**CONTEXT AND OBJECTIVE:**

Addiction to work is one of the new behavioral phenomena present in organizations and it is characterized by excess work and compulsion to work. This phenomenon may give rise to different sicknesses and may affect different professionals, including doctors. Thus, the aims of this study were to analyze the factorial validity and internal consistency of the Dutch Work Addiction Scale (DUWAS); to evaluate the prevalence of addiction to work among doctors in the state of Paraíba; and to investigate factors relating to addiction to work among these doctors.

**DESIGN AND SETTING:**

This was an exploratory, descriptive cross-sectional study with a quantitative approach conducted in municipalities in the state of Paraíba.

**METHODS:**

Data were gathered between June and October 2015, by applying a questionnaire containing sociodemographic questions and the Work Addiction Scale.

**RESULTS:**

The results showed that the Work Addiction Scale has internal consistency and factorial validity and that, in the population studied, only one factor was pointed out: addiction to work. Most of the doctors were not addicted to work; among the addicts, the addiction was not excessive; and the addiction had a positive correlation with the number of shifts done and a negative correlation with age.

**CONCLUSION:**

Greater attention to this phenomenon is required and further research on this topic is needed in order to elucidate the harm caused by addiction to work in daily medical practice.

## INTRODUCTION

With a focus on greater agility and competitiveness, organizations have begun organizational policies and practices that place higher value on professionals who have dedicated more of their time to work.^1^ To meet capitalist market demands, professionals tend to work to exhaustion in order to reach organizational targets and consequently obtain desirable professional success. Thus, work tends to be the main reason to live and exist, which creates the new phenomenon of workaholism, i.e. addiction to work^1^ or working excessively.^2^^-^^3^

Addiction to work may be considered to be one of the most recent behavioral vices, although no addiction-inducing substance is present.^3^^-^^4^ It is a form of psychosocial damage characterized by working excessively, because of an irresistible need or wish to work more and more^5^. Thus, it involves compulsion to work.^6^

These phenomena may be subjectively experienced as loss of control, in which the work addict continues working despite the known negative consequences, which are similar to those relating to psychological, physical and social disorders.^7^ The consequences of addiction to work include cardiovascular and dermatological problems, cephalalgias, myalgias, sexual disorders,^8^ depression, high-level anxiety^9^ and poor satisfaction with life and with work performance.^10^

Because of the severe health complications that addiction to work causes, it has received great attention from researchers.^11^ However, research on this topic has been hindered by a lack of consensus regarding the definition of this term and how it is evaluated.^1^^,^^7^ Moreover, there is poor knowledge about the number and kind of people affected by addiction to work and insufficient research with relevant samples.^7^

Addiction to work in the Brazilian context, factors relating to this and assessments of its consequences have been little explored in the Brazilian scientific literature. This shows that there is a need for greater scientific production concerning this topic.

Regarding the characteristics that describe addiction to work, this problem can be correlated with medical practice. Thus, it becomes relevant to evaluate the extent to which doctors may be vulnerable to addiction to work and the factors that might be related to this phenomenon.

In addition to academic relevance, the current study may provide practical contributions through increasing the knowledge of addiction to work and its correlations in Brazil. Specifically, through knowing to what extent physicians are work addicts, strategies to help these professionals improve their health and quality of life may be deployed.

## OBJECTIVES

The study aimed to analyze the factorial validity and internal consistency of the Dutch Work Addiction Scale (DUWAS); to evaluate addiction to work among doctors in the state of Paraíba; and to investigate factors relating to addiction to work among these doctors.

## METHODS

This was an exploratory analytical cross-sectional study with a quantitative approach. The population consisted of doctors in the state of Paraíba, Brazil, in the municipalities of João Pessoa, Pombal, Guarabira, Bayeux, Santa Rita, Cabedelo, Campina Grande, Sousa, Cajazeiras, Monteiro, Itaporanga, Piancó, Catolé do Rocha, Belém do Brejo dos Santos, São Bento and Patos. This study was approved by the Ethics Committee for Research on Human Beings of Lauro Wanderley University Hospital, Federal University of Paraíba. Data collection was performed only after approval had been obtained from this committee, under the number CAAE 39156114.0.0000.5183.

The sample size was calculated based on the number of physicians registered at the Regional Medical Council of Paraíba (6,100) by using a simple random sampling plan, considering a margin of error of 3% and a confidence level of 95%. Thus, the sample size was equal to 909. As it was possible to interview more physicians than the total of sample size calculated, the final sample size was 1,110 physicians.

Doctors from Sertão, Borborema, Agreste and Mata were selected to ensure the representativeness of the sample selection. It needs to be highlighted that in choosing these municipalities for inclusion, the number of doctors present at these locations was taken into consideration, because in some places there was either minimal presence of professionals or even none at all. These physicians met the following inclusion criteria: they were active during the data-gathering period; they signed a free and informed consent statement; and they were interested in participating in the study and did so willingly.

Data were gathered between June and October 2015. Sociodemographic information (age, sex, race, marital status, income, religion and number of shifts) and data relating to the Work Addiction Scale were sought. Each work shift was considered to consist of 12 working hours.

The main researcher was directly responsible for applying the questionnaire and coordinating the corresponding activities, counting on cooperation from other previously trained researchers. The average time taken to gather data from each individual was 20 minutes.

The Dutch Work Addiction Scale (DUWAS) was originally created by Schaufeli et al.^12^^,^^13^^,^^14^ It is composed of 7 items (e.g. “I am usually busy”; “I have many issues under my control”; and “I feel guilty when I am not doing anything else”). The questions are answered on a four-point scale, ranging from never (1) to every day (4). These aim to show how the person feels at work, i.e. how much he experiences. Theoretically, this measurement covers two main dimensions: compulsive work and excessive work.

The Brazilian version adapted by Carlotto and Miralles^15^ follows the same instructions and response format. However, it contains 10 items: 5 for each dimension, which are answered using a four-point scale (on which 1 = never and 4 = every day). The results from this adaption have provided justifications for using this scale, including a goodness-of-fit index = 0.95, comparative fit index = 0.98, root mean square error of approximation = 0.04 and internal consistency in which the minimum Cronbach’s alpha exceeded the cutoff point of 0.70.

Because we did not find any data relating to measurement of addiction to work among doctors, we decided to take into consideration not only the Portuguese version of the scale but also exploratory analyses that aimed to identify the underlying (componential) factorial structure.

The mean point on the scale (2.5) was used to consider whether addiction to work was present, chosen as the mean between the two cut-off values proposed by Schaufeli.^12^ Thus, the doctors whose mean score was greater than or equal to 2.5 were considered to be addicted to work. Those whose mean scores were less than 2.5 were not considered to be addicted to work.

Data were originally formatted using the Microsoft Excel software and were then converted to .sav format in the Statistical Package for the Social Sciences (SPSS) (version 18). Descriptive statistics, e.g. frequency distribution, mean and standard deviation, were produced. These were useful for ascertaining the profile of the sample participants. In addition, we also calculated the following: Kaiser-Meyer-Olkin (KMO) criterion, based on measurement of the adequacy of the factorial analysis; Bartlett sphericity test, which determines whether correlations exist among the variables; Cattel criterion, which presents the most important variables in the factorial analysis in ascending order; Cronbach’s alpha, which evaluates instrument consistency; correlation tests to evaluate addiction to work and demographic variables; mean comparison tests (t tests); and association tests (chi-square and Fisher). The significance level was taken to be 5% (P < 0.05).

## RESULTS

Although 1,110 physicians were initially interviewed at hospitals, in their shifts and healthcare centers, only 1,108 were included in the final sample: 2 were excluded due to incomplete answering of the questionnaire, making it impossible to perform correlation analysis. The majority of the participants were male (53.30%). The largest proportion of the individuals (27.17%) aged between 31 and 40 years, followed by participants between 51 and 60 years (20.40%). A minority was over 60 years of age (15.79%). Most participants stated that their monthly salary was seven or more minimum wages (92.50%) (Table 1).

**Table 1.**
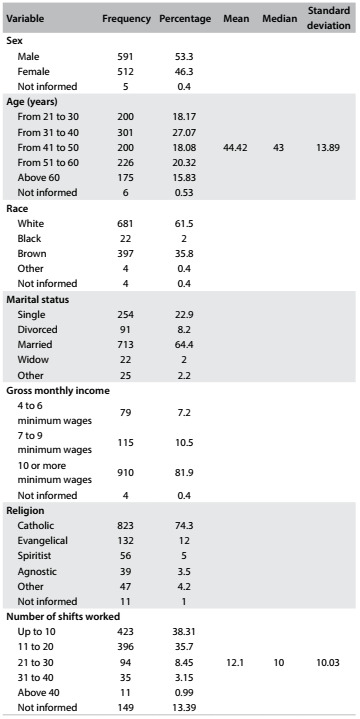
Doctors’ distribution according to sex, age, race, marital status, income, religion and number of shifts worked. Paraíba (PB), 2016 (n = 1108)

The largest proportion of the doctors performed 10 shifts (each with 12 working hours) per month (38.18%), followed by those who did 11 to 20 shifts (35.74%). The average was of 12.10 shifts per month (standard deviation, SD = 10.03). A very small proportion (3.16%) stated that they did 31 shifts or more.

### Parameters for addiction to work

As a first step, we decided to check the adequacy of the factorial analyses through an inter-item correlation matrix. The KMO (0.88) and Barlett sphericity test results [χ² (45) = 3831.36; P < 0.001] showed that these analyses were adequate for the data. Thus, we opted to perform principal component analysis (PCA).

**Figure 1.**
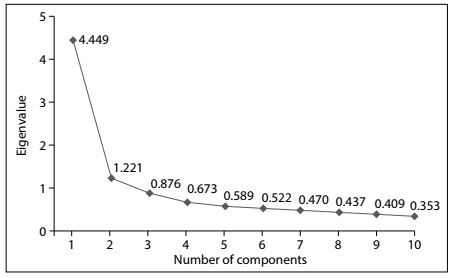
Distribution of eigenvalues on the work addiction scale.

The analyses showed that there were two components with eigenvalues greater than one (4.45 and 1.22; Kaiser criterion) that together explained 56.7% of the total variance. Figure 1 shows the graphical distribution of the eigenvalues, which is a more consistent criterion for defining the number of factors to be taken out (Cattel criterion).

As can be seen from Figure 1, the first component is evident, standing out from the others. However, regarding the other components, no relevant difference was observed; the second component is practically aligned with the others, thus suggesting that its presence may not be so clear. Because of these findings, it was considered that only one component existed, thus defining its extraction.

It was found that the saturations (factorial charges) were substantially consistent, ranging from 0.60 (item 10: “It is difficult to relax when I am not working”) to 0.70 (item 6: “I dedicate more time to work than to my friends, hobbies or pleasant activities”). Thus, it seems plausible to accept that there was only one dimension for addiction to work, which presented the eigenvalue of 4.45 and explained 44.5% of the total variance. The internal consistency (Cronbach’s alpha) of this group of items for measuring the same construct was 0.86, thus emphasizing its pertinence. Likewise, the homogeneity coefficient, i.e. the mean inter-item correlation, was 0.38. This ranged from 0.23 (item 9 with items 1 and 4) to 0.61 (items 7 and 9).

In summary, it can be accepted that the Work Addiction Scale evaluated a general factor gathering together the factorial validity and evidence for internal consistency in the group studied here, which can be simply named “addiction to work”. All the results from these analyses thus showed that the general score from the Work Addiction Scale, obtained through summing its 10 items, could be considered to be the sole variable. This could then be analyzed.

### Addiction to work among doctors

In the present analysis, most of the physicians were not addicted to work (610 in total). From the average scores for addiction to work in each group (addicts and non-addicts), it was seen that the non-addicts had an average score of 1.95 (SD = 0.32), and the addicts had a score of 2.50 (SD = 0.37). Thus, it could be confirmed that even in the group of addicts, the addiction was not strong, given that the addiction score was only 2.5.

### Factors relating to addiction to work

Addiction to work was positively and weakly correlated with the number of shifts worked (r = 0.20; P < 0.001); and it showed a negative, weak correlation with age (r = -0.20; P < 0.0001). Income did not show any significant correlation with addiction to work (r = -0.01; P > 0.05).

It was not possible to show any association between categorized addiction to work and the variables of sex, race, marital status and religion, with the confidence level taken to be 95%.

From these findings, it seems evident that addiction to work was influenced by the number of shifts worked and the doctors’ ages. On this basis, the younger the doctor was, the greater the chances were of being addicted.

## DISCUSSION

The results from this study showed that the Work Addiction Scale has internal consistency and factorial validity, and that it evaluates a single general factor that can be named “addiction to work”, which is more precise than bi-dimensional evaluation. These findings are contrary to the results found by Carlotto and Miralles,^15^ who found that bi-dimensional evaluation of compulsion to work and excessive work was coherent.

In an evaluation on the factorial validity and internal consistency of the Brazilian Work Addiction Scale, it was observed that compulsion to work and excessive work were related but independent dimensions.^15^ However, the group studied and the research locality differed from those of the present study, which may have contributed towards the divergence of these results.

Nevertheless, the study in which the factorial structure of the 10-item version of the Dutch Work Addiction Scale (DUWAS) was investigated demonstrated that this measurement could be applied rapidly and had good factorial validity for all the professions and nations measured. Moreover, it has showed that the DUWAS-10 subdimensions were highly correlated, thus making it possible to use them as a one-dimensional measurement for addiction to work.^16^

That study^16^ also showed that the degree of addiction to work can be assessed through four dimensions that are subdimensions of compulsion to work and excessive work: “working frantically” and “working long hours” to explain excessive work and “obsessive work drive” and “unease if not working” to explain compulsion to work.

In the light of these variabilities, further studies replicating the results obtained through using workers in a diversity of professional categories, in different regions of Brazil and in dissimilar organizational and sociocultural contexts, are recommended. These would take into consideration the specificity of each group.^15^

The current study showed that, although present, the addiction to work was not strong. The fact that most of the professionals evaluated only had up to 10 monthly shifts may have influenced the scores for addiction to work. The addicts dedicated many hours to their professional practice, but this was not a constant factor in the group studied.

In another study, doctors considered that their work was one of the most important things in their lives, and that it fulfilled their economic and emotional needs and their pleasure and status, self-satisfaction and self-valorization. These factors motivated them, were complementary and made life more attractive, and may have influenced their non-addiction to work.^17^

A qualitative study involving eight doctors showed that they did not fulfill the characteristics of workaholism, although the interviewees worked exhaustingly long hours and had difficulties regarding taking vacations, having leisure time, being with their families and disassociating their thoughts from their work. However, on certain occasions, some traces were observed, but they were not significant enough to affirm that these doctors were workaholics.^17^

A case study using a quantitative approach analyzed a sample of 25 collaborators within a medical healthcare cooperative, using a self-reporting questionnaire consisting of 24 questions. The results showed that there were many workers who might become workaholics, but that only four of them were considered to be workaholics.^1^

However, this does not mean that these professionals are also worklovers*.* A worklover is a person who is always satisfied with his work, and this is not a common occurrence among healthcare professionals, including physicians.^17^

An analysis on 2,115 resident Dutch physicians showed that unfavorable work conditions, work overload, conflicts, overwork, presentism and burnout syndrome were related to addiction to work. In addition, it showed that addicts recovered less easily, felt less fulfilled, were unhappy and had a colder relationship with their patients. Thus, the combination of compulsion to work and excessive work was detrimental both to doctors and to the institution in which they work.^18^

A study carried out among doctors in the northeastern states of Brazil and published by the Federal Medicine Council showed that doctors in Paraíba mostly considered that their working conditions were fair or good, and that they were satisfied with the work that they were doing. They also believed that their working day and pay had improved in relation to previous years.^19^ These factors may contribute towards a lack of addiction to work.

In view of these favorable working conditions and reasonable salaries, plus low wear and tear relating to transportation within the doctors’ municipalities, i.e. without the hectic pace of larger centers (which means that they do not waste so much time on commuting), a healthy routine encompassing work, home and family becomes established. This favors biopsychosocial and economic balance among physicians in Paraíba and frees them from being absorbed into addiction to work. Moreover, because they are close to the countryside, they can enjoy moments of leisure, when they have become established.

Addiction to work among doctors was correlated positively with the number of shifts worked and negatively with age in the present study. Thus, the more shifts worked monthly and the younger the doctor was, the greater the addiction to work was. Workaholics tend to work compulsively and to work more than what is required within their work environment, for greater financial return and professional status and because of the organizational culture.^20^ These factors may influence the relationship between the number of shifts and work addiction.

This study showed also that intense dedication to work involved a great number of hours taken to conclude activities. If different demands are under these individuals’ control, more time is required and this means greater addiction to work.^21^ The fact that younger physicians need to achieve professional status and excel in the job market may make them work intensively, with longer working hours, thus dedicating more time to work and giving up leisure time. This increases their chances of developing addiction to work. However, it has been suggested that young workers who do not have a family are more likely to gain professional achievement and receive little social criticism if they work excessively, in comparison with older workers who are married and have children. The latter attributes contribute towards decreasing addiction to work.^22^ A study conducted on a Portuguese group showed that younger people seem to work less excessively than older ones.^23^ In that study, there was no significant correlation between addiction to work and the income variable, or even any associations with the variables of sex, race, marital status and religion.

It is important to point out that, in many cases, low income threatens workers’ personal, family and social stability and, in searching for better work conditions, they dedicate more time to work, which may make them become workaholics. However, in comparing medical activities with other professions in Brazil, it needs to be highlighted that medical activity is well remunerated, and that this may influence the lack of correlation between addiction and income.

The findings from the present study were similar to those from a previous study that showed that there was no relationship between the dimensions of addiction to work and the variables of income, sex and marital status, among others.^24^ A study conducted among academic groups showed that addiction to work had a negative correlation with age, wages and marital status and a positive correlation with the kind and size of the family.^25^

Addiction involves behavioral vices without substance, and it is necessary to differentiate this from real addiction to work, since this behavior may generate mental, physical, social and family consequences, besides affecting work performance.^26^ Different people have considered work to be the most important factor in their lives, for different reasons such as: the need to survive, professional and personal fulfilment or even financial reasons. Consequently, some people have shown greater predisposition towards developing addiction to work.^1^ So far, no treatment for addiction to work has been proposed, and it is therefore necessary to conduct further studies in this area.^27^

Moreover, there is a need for additional studies in relation to considering work as one of the predictors of sickness, especially nowadays, when work is considered to be a privilege, and not a personal right. Only advances of science within this field may provide the credibility that is required for there to be any kind of influence on public policies for workers and any contribution towards international scientific outcomes relating to addiction to work.^15^

This study has limitations, since the findings cannot be generalized to the medical profession in general because only one Brazilian state was analyzed. In addition to this, the analysis only considered the relationship between addiction and the variables of sex, age, income, race, marital status, religion and working hours. Other studies evaluating relationships with other variables such as presence of children, length of time as a professional and leisure time are also necessary.

This phenomenon requires closer attention. Further research that can elucidate the damage caused by addiction to work within daily medical practice and which can create strategies for diminishing addiction to work and its consequences needs to be developed to investigate the presence of addiction to work among doctors in other Brazilian regions and the factors that influence it.

## FINAL REMARKS

In keeping with the traditional characteristics of Northeasterners, of love for the land and contact with nature, the bucolic life of rural areas is an incentive for physicians, once they have become established, to acquire the hobby of spending weekends enjoying the countryside or farms near their homes and having moments of pleasure, which contribute towards increasing their wellbeing. The pleasure of being together with their relatives, and with the soil, animals and plants, relieves the tension of the day-to-day routine and is an excellent impediment to work addition.

As expected, the higher the number of shifts worked was, the greater the addiction to work was, but the greater the age was, the less the addiction was. Since addiction is due to repetition, it is believed that when doctors take on more and more shifts, they get into a vicious cycle and become liable to take up the repetitive act of working more and more. They thus compromise their good sense of knowing how to impose limits and refuse additional job offers, and consequently let themselves be swallowed up by the addiction.

Regarding the predominance of addiction in the younger age group, it is likely to be due to the natural impulses of youth, consisting of “I want everything”, “I can do everything” and “I crave everything”. It may even be due to a certain degree of ambition to quickly ascend socially and economically through the product of their work, i.e. the more they work, the more they earn and the richer they can become.

Another aspect of this might be due to the long duration of medical school. Because medical courses are full-time, they do not allow young people to enter profitable employment during the six years of the course, plus another four to six more years of medical residency. When doctors finally become free to enter the job market, the will to “roll up the sleeves and get to work” manifests itself more intensely, and the long-held desire to “make money” is shown, sometimes even to an exaggerated extent.

## CONCLUSION

The work addiction scale that best suited the population studied here considered excessive work and compulsion to work to be dependent variables. Therefore, the scale is unifactorial.

Most of the physicians evaluated were not addicted to work, and, even in the group of addicts, the addiction was not strong, given that the addiction score was only 2.5. This may have been because these physicians considered that their working conditions were favorable and because they were satisfied with what they were doing.
